# The complete genome sequence of the rumen methanogen *Methanobacterium formicicum* BRM9

**DOI:** 10.1186/1944-3277-9-15

**Published:** 2014-12-08

**Authors:** William J Kelly, Sinead C Leahy, Dong Li, Rechelle Perry, Suzanne C Lambie, Graeme T Attwood, Eric Altermann

**Affiliations:** 1 Rumen Microbiology, Animal Nutrition and Health, AgResearch Limited, Grasslands Research Centre, Tennent Drive, Private Bag 11008, Palmerston North 4442, New Zealand; 2 New Zealand Agricultural Greenhouse Gas Research Centre, Grasslands Research Centre, Tennent Drive, Private Bag 11008, Palmerston North 4442, New Zealand; 3 Riddet Institute, Massey University, Palmerston North 4442, New Zealand

**Keywords:** Methanogen, Methane, Ruminant, *Methanobacterium formicicum*

## Abstract

*Methanobacterium formicicum* BRM9 was isolated from the rumen of a New Zealand Friesan cow grazing a ryegrass/clover pasture, and its genome has been sequenced to provide information on the phylogenetic diversity of rumen methanogens with a view to developing technologies for methane mitigation. The 2.45 Mb BRM9 chromosome has an average G + C content of 41%, and encodes 2,352 protein-coding genes. The genes involved in methanogenesis are comparable to those found in other members of the *Methanobacteriaceae* with the exception that there is no [Fe]-hydrogenase dehydrogenase (Hmd) which links the methenyl-H4MPT reduction directly with the oxidation of H_2_. Compared to the rumen *Methanobrevibacter* strains, BRM9 has a much larger complement of genes involved in determining oxidative stress response, signal transduction and nitrogen fixation. BRM9 also has genes for the biosynthesis of the compatible solute ectoine that has not been reported to be produced by methanogens. The BRM9 genome has a prophage and two CRISPR repeat regions. Comparison to the genomes of other *Methanobacterium* strains shows a core genome of ~1,350 coding sequences and 190 strain-specific genes in BRM9, most of which are hypothetical proteins or prophage related.

## Introduction

Ruminants have evolved an efficient digestive system in which microbes ferment the plant material that constitutes the animal’s diet to produce short chain fatty acids, principally acetic, propionic and butyric acids, and other products [1]. This fermentation is carried out by a complex microbial community which includes bacteria, ciliate protozoa, anaerobic fungi, and methanogenic archaea, and has been the focus of numerous studies. The role of the methanogenic archaea in the rumen environment is important as they use hydrogen (H_2_) derived from microbial fermentation as their energy source and combine it with carbon dioxide (CO_2_) to form methane (CH_4_), which is belched from the animal and released to the atmosphere. Other fermentation end-products including formate and methyl-containing compounds can also be substrates for methanogenesis [2].

Methane is a potent greenhouse gas contributing to global climate change, and ruminant derived CH_4_ accounts for about one quarter of all anthropogenic CH_4_ emissions [3]. Development of strategies to reduce CH_4_ emissions from farmed animals are currently being investigated, and methanogen genome sequence information has already been used to inform CH_4_ mitigation strategies based on vaccines and small-molecule inhibitors [4,5]. CH_4_ mitigation technologies should target features that are conserved across all rumen methanogens, and be methanogen-specific so that other rumen microbes can continue their normal digestive functions. To address this we are sequencing the genomes of cultures that represent the phylogenetic diversity of rumen methanogens to define their conserved features as targets for developing CH_4_ mitigation technologies [4,6,7], and to understand their role in the rumen environment, and interactions with other members of the rumen microbiome.

## Organism information

**Figure 1:**
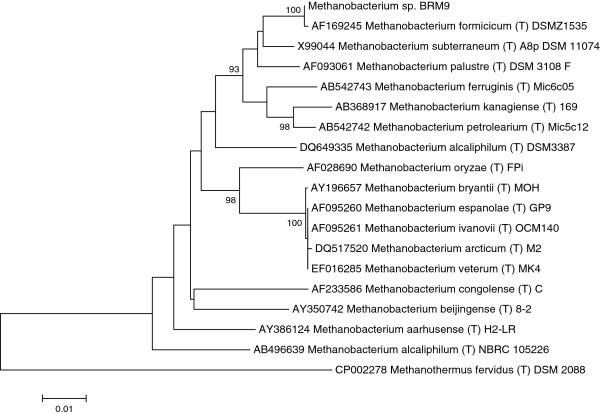
**Phylogenetic tree showing the position of *Methanobacterium *sp.** BRM9 relative to type strains of other *Methanobacterium* species*.* The strains and their corresponding accession numbers are shown. The evolutionary history was inferred using the Neighbor-Joining method [14] with *Methanothermus fervidus* used as an outgroup. The optimal tree with the sum of branch length = 0.34833139 is shown. The percentage of replicate trees (>90%) in which the associated taxa clustered together in the bootstrap test (1000 replicates) are shown next to the branches [15]. The tree is drawn to scale, with branch lengths in the same units as those of the evolutionary distances used to infer the phylogenetic tree. The evolutionary distances were computed using the Kimura 2-parameter method [16] and are in the units of the number of base substitutions per site. The analysis involved 19 nucleotide sequences. All positions containing gaps and missing data were eliminated. There were a total of 1168 positions in the final dataset. Evolutionary analyses were conducted in MEGA5 [17].

*Methanobacterium* sp. BRM9 was isolated from the rumen of a New Zealand Friesan cow grazing a ryegrass/clover pasture [8]. It was described as a Gram positive non-motile, short rod which becomes a long, irregular rod at later growth stages. It is able to grow and produce methane from formate and H_2_/CO_2_, but not from acetate, alcohols or methylamines. Growth occurred over a wide temperature range (25–45°C) and at pH 6–8. Rumen fluid was required for growth. The 16S rRNA from BRM9 is 99.8% similar to the *M. formicicum* type strain DSM 1535 [Figure 1] which was isolated from a sewage sludge digester [9,10] and as such BRM9 can be considered as a strain of *M. formicicum. M. formicicum* is found at high densities in anaerobic digesters and freshwater sediments, and has previously been isolated from the rumen [11], although *Methanobacterium* species only occur at low density in this environment [2]. Isolates have also been obtained as endosymbionts of anaerobic amoebae and ciliate protozoa species. Electron microscopic studies of *M. formicicum* show a long rod shaped morphology, and cells characterized by numerous cytoplasmic membrane bodies believed to be formed by invagination of the cell membrane [12,13]. Characteristics of *M. formicicum* BRM9 are shown in Table 1 and Additional file 1: Table S1

**Table 1:** **Classification and general features of *Methanobacterium formicicum *** **BRM9**

**MIGS ID**	**Property**	**Term**	**Evidence code**^**a**^
	Current classification	Domain: *Archaea*	TAS [18]
		Phylum: *Euryarchaeota*	TAS [19]
		Class: *Methanobacteria*	TAS [20,21]
		Order: *Methanobacteriales*	TAS [22-24]
		Family: *Methanobacteriaceae*	TAS [25]
		Genus: *Methanobacterium*	TAS [23]
		Species: *Methanobacterium formicicum* strain BRM9	TAS [8]
	Gram stain	Positive	TAS [8]
	Cell shape	Rod	TAS [8]
	Motility	No	TAS [8]
	Sporulation	No	IDA
	Temperature range	25-45°C	TAS [8]
	Optimum temperature	38°C	TAS [8]
	Carbon source	CO_2_, Acetate	IDA
	Energy source	H_2_ + CO_2_, formate	TAS [8]
	Terminal electron receptor	CO_2_	IDA
MIGS-6	Habitat	Bovine rumen	TAS [8]
MIGS-6.3	Salinity	not reported	
MIGS-22	Oxygen	Strict anaerobe	IDA
MIGS-15	Biotic relationship	Symbiont of ruminants	TAS [8]
MIGS-14	Pathogenicity	Not known as a pathogen	NAS
MIGS-4	Geographic location	Palmerston North, New Zealand	IDA
MIGS-5	Sample collection time	Not reported	
MIGS-4.1	Latitude	Latitude: −40.35 (40°21′00″S)	IDA
MIGS-4.2	Longitude	Longitude: +175.61 (175°36′36″E)	IDA
MIGS-4.3	Depth	Not reported	
MIGS-4.4	Altitude	30 m	IDA

^a^Evidence codes – TAS: Traceable Author Statement; IDA: Inferred from Direct Assay; NAS: Non-traceable Author Statement (i.e., not directly observed for the living, isolated sample, but based on a generally accepted property for the species, or anecdotal evidence) [26].

## Genome sequencing information

### Genome project history

*Methanobacterium formicicum* BRM9 was selected for genome sequencing on the basis of its phylogenetic position relative to other methanogens belonging to the family *Methanobacteriaceae*. Table 2 presents the project information and its association with MIGS version 2.0 compliance [27].

**Table 2:** Project information

**MIGS ID**	**Property**	**Term**
MIGS-31	Finishing quality	high-quality, closed genome
MIGS-28	Libraries used	3 Kb mate paired-end library
MIGS-29	Sequencing platforms	454 GS FLX, Titanium chemistry
MIGS-31.2	Fold coverage	97x
MIGS-30	Assemblers	Newbler
MIGS-32	Gene calling method	Glimmer and BLASTX
	Genome Database release	October 2, 2014
	Genbank ID	CP006933
	Genbank Date of Release	October 2, 2014
	GOLD ID	Gp0007264
	Project relevance	Ruminant methane emissions

### Growth conditions and DNA isolation

BRM9 was grown in BY medium [28] with added SL10 Trace Elements solution (1 ml added l^−1^) [29], Selenite/Tungstate solution (final concentration of selenite and tungstate are 3 and 4 μg l^−1^ respectively), [30] and Vitamin 10 solution (0.1 ml added to 10 ml culture before inoculation) [6]. H_2_ was supplied as the energy source by pumping the culture vessels to 180 kPa over pressure with an 80:20 mixture of H_2_:CO_2_. Genomic DNA was extracted from freshly grown cells using a modified version of a liquid N_2_ and grinding method [31]. Briefly, BRM9 cultures were harvested by centrifugation at 20,000 × *g* for 20 min at 4°C and cell pellets combined into 40 ml Oakridge centrifuge tubes and frozen at −80°C. The frozen cell pellets were placed in a sterile, pre-cooled (−85°C) mortar and ground to a powder with periodic addition of liquid N_2_. Buffer B1 (5 ml Qiagen Genomic-Tip 500 Maxi kit, Qiagen, Hilden, Germany) containing RNase (2 μg ml^−1^ final concentration) was added to the powdered cell pellet to create a slurry which was then removed to a 15 ml Falcon tube. An additional 6 ml of B1 buffer was used to rinse the remaining material from the mortar and pestle and combined with the cell slurry, which was then treated following the Qiagen Genomic-Tip 500/G Maxi kit instructions. Finally, the genomic DNA was precipitated by the addition of 0.7 vol isopropanol, and collected by centrifugation at 12,000 × *g* for 10 min at room temperature. The supernatant was removed, and the DNA pellet was washed in 70% ethanol, re-dissolved in TE buffer (10 mM Tris–HCl, 1 mM EDTA pH 7.5) and stored at −20°C until required.

## Genome sequencing and assembly

The complete genome sequence of BRM9 was determined using pyrosequencing of 3Kb mate paired-end sequence libraries using a 454 GS FLX platform with Titanium chemistry (Macrogen, Korea). Pyrosequencing reads provided 97× coverage of the genome and were assembled using the Newbler assembler version 2.0 (Roche 454 Life Sciences, USA). The Newbler assembly resulted in 85 contigs across 9 scaffolds. Gap closure was managed using the Staden package [32] and gaps were closed using additional Sanger sequencing by standard and inverse PCR based techniques. A total of 219 additional reactions were used to close gaps and to improve the quality of the genome sequence to ensure correct assembly and to resolve any remaining base-conflicts. Assembly validation was confirmed by pulsed-field gel electrophoresis as described previously [6], using the enzyme AscI which cuts the BRM9 chromosome at 6 sites.

### Genome annotation

A GAMOLA/ARTEMIS [33,34] software suite was used to manage genome annotation. Protein-encoding open reading frames (ORFs) were identified using the ORF-prediction program Glimmer [35] and BLASTX [36,37]. A manual inspection was performed to verify or, if necessary, redefine the start and stop codons of each ORF. Assignment of protein function to ORFs was performed manually using results from the following sources; BLASTP [36] to both a non-redundant protein database provided by the National Centre for Biotechnology Information (NCBI) [38] and Clusters of Orthologous Groups (COG) database [39]. HMMER [40] was used to identify protein motifs to both the PFAM [41] and TIGRFAM [42] libraries. TMHMM [43,44] was used to predict transmembrane sequences, and SignalP, version 4.1 [45] was used for the prediction of signal peptides. Ribosomal RNA genes were detected on the basis of BLASTN searches to a custom GAMOLA ribosomal database. Transfer RNA genes were identified using tRNAscan-SE [46]. Miscellaneous-coding RNAs were identified using the Rfam database [47] utilizing the INFERNAL software package [48]. The genome sequence was prepared for NCBI submission using Sequin [49]. The adenine residue of the start codon of the Cdc6-1 replication initiation protein (BRM9_0001) gene was chosen as the first base for the BRM9 genome. The nucleotide sequence of the *Methanobacterium formicicum* BRM9 chromosome has been deposited in Genbank under accession number CP006933.

## Genome properties

The genome of *Methanobacterium formicicum* BRM9 consists of a single 2,449,988 basepair (bp) circular chromosome with an average G + C content of 41%. A total of 2,418 genes were predicted, 2,352 of which were protein-coding genes, representing 83% of the total genome sequence. A putative function was assigned to 1,715 of the protein-coding genes, with the remainder annotated as hypothetical proteins. The properties and statistics of the genome are summarized in Tables 3, 4 and 5. The BRM9 genome has a 37 Kb prophage (BRM9_1642-1689) with several genes that have best matches to those from other prophage. The phage ORFs are flanked by 22 bp sequences indicative of *attL* and *attR* sites. In addition there are several CRISPR genes associated with two CRISPR repeat regions of 7178 and 11914 bp, as well as the components of a type I restriction-modification system.

**Table 3:** Summary of genome

**Label**	**Size (Mb)**	**Topology**	**INSDC identifier**
Chromosome	2.45	Circular	CP006933

**Table 4:** Nucleotide content and gene count levels of the genome

**Attribute**	**Genome (total)**	
	**Value **	**% of total**^**a**^
Size (bp)	2,449,987	100.00
G + C content (bp)	1,012,813	41.34
Coding region (bp)	2,028,429	82.79
Total genes^b^	2,418	100.00
RNA genes	52	2.15
Protein-coding genes	2352	97.27
Genes assigned to COGs	1,715	70.93
Genes with signal peptides	95	3.93
Genes with transmembrane helices	573	23.70

^a^The total is based on either the size of the genome in base pairs or the total number of protein coding genes in the annotated genome.

^b^Also includes 14 pseudogenes.

**Table 5:** Number of genes associated with the 25 general COG functional categories

**Code**	**Value**	**% of total**^**a**^	**Description**
J	148	6.29	Translation
A	1	0.04	RNA processing and modification
K	104	4.42	Transcription
L	93	3.95	Replication, recombination and repair
B	4	0.17	Chromatin structure and dynamics
D	10	0.42	Cell cycle control, mitosis and meiosis
Y	-	-	Nuclear structure
V	37	1.57	Defense mechanisms
T	72	3.06	Signal transduction mechanisms
M	64	2.72	Cell wall/membrane biogenesis
N	5	0.21	Cell motility
Z	-	-	Cytoskeleton
W	-	-	Extracellular structures
U	13	0.55	Intracellular trafficking and secretion
O	55	2.34	Posttranslational modification, protein turnover, chaperones
C	187	7.95	Energy production and conversion
G	51	2.17	Carbohydrate transport and metabolism
E	121	5.14	Amino acid transport and metabolism
F	50	2.12	Nucleotide transport and metabolism
H	93	3.95	Coenzyme transport and metabolism
I	30	1.27	Lipid transport and metabolism
P	92	3.91	Inorganic ion transport and metabolism
Q	26	1.10	Secondary metabolites biosynthesis, transport and catabolism
R	270	11.47	General function prediction only
S	189	8.04	Function unknown
-	637	27.08	Not in COGs

^a^The total is based on the total number of protein coding genes in the annotated genome.

## Insights from the genome

The genes involved in methanogenesis are comparable to those found in other members of the *Methanobacteriaceae* with the exception that there is no [Fe]-hydrogenase dehydrogenase (Hmd) which links the methenyl-H4MPT reduction directly with the oxidation of H_2_. BRM9 has the methyl coenzyme M reductase II genes (*mrt*AGDB, BRM9_2153-2156), unlike *Methanobrevibacter* strains M1 and AbM4 [6,7]. BRM9 has a cysteinyl-tRNA synthetase (*cys*S), but also encodes the alternative tRNA-dependent cysteine biosynthesis pathway (*sep*S/*psc*S) found in *Methanocaldococcus jannaschii* and other methanogens [50] but not in *Methanobrevibacter* sp. BRM9 also has a carbon monoxide dehydrogenase/acetyl-coenzyme A synthase (CODH/ACS, or Cdh) to fix CO_2_ and form acetyl-CoA, and several acetyl-CoA synthetases one of which is located next to a possible acetate permease (BRM9_1255). Like many other methanogens, the CODH/ACS genes in BRM9 are found in a single cluster (BRM9_0795-0801). There is also a NAD-dependent malic enzyme (BRM9_2358) able to catalyse the oxidative decarboxylation of malate to form pyruvate and CO_2_. This is found in three other *Methanobacterium* strains (MBC34, PP1, SWAN-1) but not in other members of the *Methanobacteriaceae*.

The cell walls of members of the *Methanobacteriaceae* consist of pseudomurein and while the pathway for pseudomurein biosynthesis and its primary structure have been elucidated the enzymes involved have not been characterized. The predicted pseudomurein biosynthesis genes are similar to those found in *Methanobrevibacter* species [6], but there are differences in the other cell wall glycopolymers. BRM9 has several proteins with multiple copies of the PMBR domain (Pfam accession PF09373) predicted to be involved in binding to pseudomurein. There are four clusters of genes involved in polysaccharide biosynthesis and two oligosaccharyl transferases, but BRM9 does not have homologues of neuA/neuB found in other methanogen strains including *M. formicicum* DSM 3637 [51]. BRM9 has fewer cell surface proteins than do *Methanobrevibacter* species, and these contain a range of different repeat domains.

Compared to the rumen *Methanobrevibacter* species BRM9 has a much larger complement of activities involved in oxidative stress response with a superoxide dismutase, a catalase/peroxidase and a peroxiredoxin (alkyl hydroperoxide reductase). BRM9 also has the three ectoine biosynthetic genes (*ect*ABC, BRM9_2205-2207) that encode production of the compatible solute ectoine that is normally found in halophilic or halotolerant organisms but has not been reported to be produced by methanogens [52]. The ectoine biosynthetic genes in BRM9 show no BLAST matches to other methanogens but have significant matches to *Dehalogenimonas lykanthroporepellens*, a dehalogenating bacterium from the phylum Chloroflexi isolated from contaminated groundwater [53]. The *ect*B and *ect*C genes also show homology to those from the rumen bacterium *Wolinella succinogenes*. Unlike the *Methanobrevibacter* species BRM9 has a large number of genes encoding components of histidine kinase/response regulator signal transduction systems. Many of these proteins include 1–5 PAS domains. These are believed to monitor changes in redox potential, oxygen, and the overall energy level of the cell [54].

The metabolism of nitrogen by BRM9 is somewhat different from *Methanobrevibacter* M1 and AbM4. BRM9 has two ammonium transporters and encodes the glutamine synthase (GS)/glutamate synthase (glutamine:2-oxoglutarate aminotransferase, GOGAT) pathway of ammonium assimilation. *Methanobacterium formicicum* has been reported to fix nitrogen [55] and BRM9 contains a *nif* operon similar to that found in *Methanococcus maripaludis* and composed of nitrogenase and nitrogenase cofactor biosynthesis genes. Nitrogen assimilation genes are regulated by NrpR which represses transcription of nitrogen fixation genes, glutamine synthase, ammonium transporters and some other genes in *M. maripaludis*[56]. NrpR binds to inverted repeat operators in the promoter regions of these genes. The inverted repeat sequence recognized is GGAAN6TTCC and occurs in BRM9 upstream from the starts of *gln*A, *nif*H, *pdx*T, *amt*1 and *amt*2.

**Figure 2:**
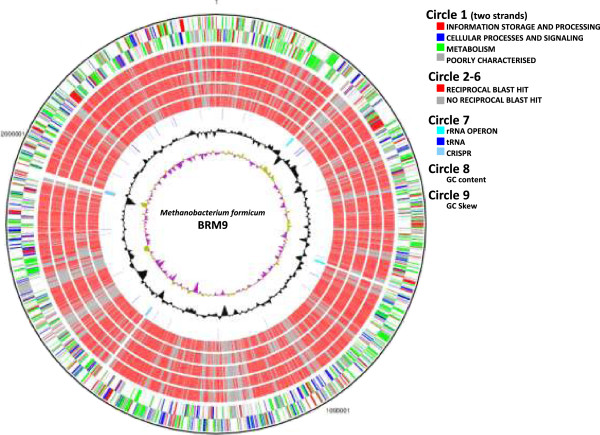
**Genome atlas of *Methanobacterium formicum *BRM9.** The circles from the outside represent: (1) forward and reverse coding domain sequences (CDS), the color coding of the CDS represent different Clusters of Orthologous Groups (COG) categories; (2) Reciprocal BLAST results with *Methanobacterium* strains MBC34; (3) PP1; (4) AL-21; (5) SWAN-1; (6) MB1; (7) rRNA, tRNA and CRISPR regions; (8) % GC plot; (9) GC skew [(GC)/(G + C)].

**Figure 3:**
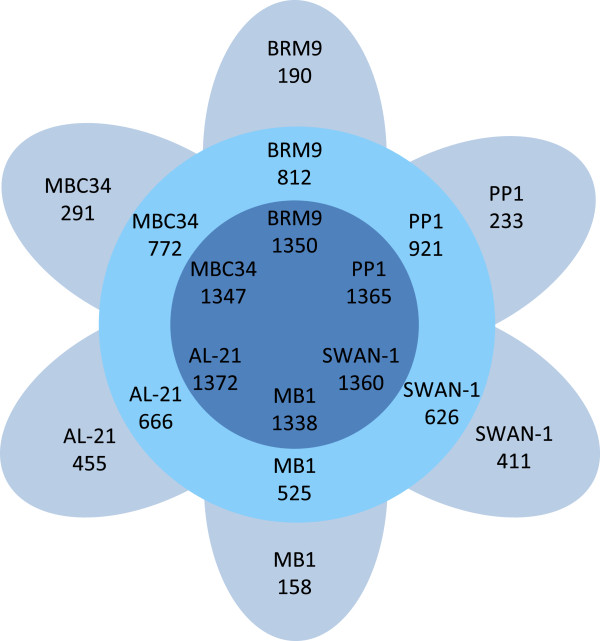
**Flower plot illustrating the number of shared and specific genes based on OrthoMCL [**[61]**] analysis of *Methanobacterium *** **genomes.**

The genome of *M. formicicum* BRM9 is compared with those of other sequenced methanogens from the genus *Methanobacterium* in Table 6. The genome atlas of *M. formicicum* BRM9 is shown in Figure 2 and indicates that the gene content of these *Methanobacterium* strains is highly similar. Comparison of the ORFeome of BRM9 with those of other sequenced *Methanobacterium* species [Figure 3] shows a core genome of ~1,350 genes. There are 190 strain-specific genes in BRM9, which include the ectoine biosynthesis genes, CRISPR and prophage-related genes as well as numerous hypothetical proteins.

**Table 6:** **Genomes of *Methanobacterium *** **species from various anaerobic environments**

**Species**	**Isolation source**	**Genome size (Mb)**	**Accession #**	**CDS**	**% GC**	**Reference**
*Methanobacterium formicicum* BRM9	Bovine rumen	2.45	CP006933	2,352	41	This report
*Methanobacterium formicicum* PP1 (DSM3637)	Free-living amoeba endosymbiont	~2.68	AMPO00000000	2,519	38	[57]
*Methanobacterium* sp. Maddingley MBC34	Coal seam formation water	~2.42	AMGN00000000	2,411	39	[58]
*Methanobacterium lacus* AL-21	Peatland [59]	2.58	CP002551	2,533	36	
*Methanobacterium paludis* SWAN-1	Peatland [59]	2.55	CP002772	2,442	36	
*Methanobacterium* sp. Mb1	Biogas plant	2.03	HG425166	2,021	40	[60]

## Conclusions

This is the first report of a genome sequence for a *Methanobacterium formicicum* strain of rumen origin. The genus *Methanobacterium* consists of mesophilic methanogens from diverse anaerobic environments, but they only constitute a small proportion of the methanogen diversity in the rumen. However, the similarity in gene content between BRM9 and strains from other environments implies that BRM9 is not particularly adapted to the rumen and may struggle in competition with the better adapted *Methanobrevibacter* species. The conserved nature of the *M. formicicum* BRM9 genes for methanogenesis, central metabolism and pseudomurein cell wall formation suggest that this species will be amenable to inhibition by the small molecule inhibitor and vaccine-based methane mitigation technologies that are being developed for the other genera of methanogens found in the rumen.

## Competing interests

The authors declare that they have no competing interests.

## Authors’ contributions

WJK, SCL, GTA, EA conceived and designed the experiments. SCL, DL, RP, SCLa performed the sequencing and assembly experiments. WJK, SCL, EA performed the annotation and comparative studies. WJK, SCL wrote the manuscript. All authors commented on the manuscript before submission. All authors read and approved the final manuscript.

## Supplementary Material

Additional file 1: Table S1 Associated MIGS record. Click here for file
