# Supplementary material for: The complete genome sequence of the rumen methanogen Methanobacterium formicicum BRM9
Source: Stand Genomic Sci. 2014 Dec 8;9:15. doi: 10.1186/1944-3277-9-15 (PMC4335013; doi:10.1186/1944-3277-9-15)
Supplement: Additional file 1: Table S1 — Associated MIGS record. [file 1944-3277-9-15-S1.doc]

**Table S1.** Associated MIGS record

| **MIGS-ID** | field name | description |
| --- | --- | --- |
| **MIGS-1** | Submit to INSDC/Trace archives |  |
| **1.1** | PID |  |
| **1.2** | Trace Archive |  |
| **MIGS-2** | MIGS CHECK LIST TYPE |  |
| **MIGS-3** | Project Name | *Methanobacterium formicicum* BRM9 |
| **MIGS-4** | Geographic Location | Palmerston North, New Zealand |
| **4.1** | Latitude | -40.35 (40°21'00"S) |
| **4.2** | Longitude | +175.61 (175°36'36"E) |
| **4.3** | Depth | NA |
| **4.4** | Altitude | 30m |
| **MIGS-5** | Time of Sample collection | Not reported |
| **MIGS-6** | Habitat (EnvO) | Cow rumen |
| **6.1** | temperature | 37-39^o^C |
| **6.2** | pH |  |
| **6.3** | salinity |  |
| **6.4** | chlorophyll |  |
| **6.5** | conductivity |  |
|  |  |  |
| **6.6** | light intensity |  |
| **6.7** | dissolved organic carbon (DOC) |  |
| **6.8** | current |  |
| **6.9** | atmospheric data |  |
| **6.10** | density |  |
| **6.11** | alkalinity |  |
| **6.12** | dissolved oxygen |  |
| **6.13** | particulate organic carbon (POC) |  |
| **6.14** | phosphate |  |
| **6.15** | nitrate |  |
| **6.16** | sulfates |  |
| **6.17** | sulfides |  |
| **6.18** | primary production |  |
| **MIGS-7** | Subspecific genetic lineage | Strain BRM9 |
| **MIGS-9** | Number of replicons | 1 |
| **MIGS-10** | Extrachromosomal elements | 0 |
| **MIGS-11** | Estimated Size | 2,449,987bp |
| **MIGS-12** | Reference for biomaterial or Genome report |  |
| **MIGS-13** | Source material identifiers | Bovine rumen contents |
| **MIGS-14** | Known Pathogenicity | Not known as a pathogen |
|  |  |  |
| **MIGS-15** | Biotic Relationship | Rumen symbiont |
| **MIGS-16** | Specific Host | Bovine |
| **MIGS-17** | Host specificity or range (taxid) | Ruminants |
| **MIGS-18** | Health status of Host | Healthy |
| **MIGS-19** | Trophic Level | Hydrogenotrophic methanogen |
| **MIGS-22** | Relationship to Oxygen | Strict anaerobe |
| **MIGS-23** | Isolation and Growth conditions | Isolated from a formate enrichment culture of a cow rumen sample |
| **MIGS-27** | Nucleic acid preparation | Freeze grinding |
| **MIGS-28** | Library construction | 3Kb Mate paired end |
| **28.1** | Library size | 237007986bp |
| **28.2** | Number of reads | 612674 |
| **28.3** | vector | NA |
| **MIGS-29** | Sequencing method | Pyrosequencing |
| **MIGS-30** | Assembly |  |
| **30.1** | Assembly method | Newbler, Staden Package |
| **30.2** | estimated error rate |  |
| **30.3** | method of calculation |  |
| **MIGS-31** | Finishing strategy |  |
| **31.1** | Status | Closed |
| **31.2** | coverage | 97x |
| **31.3** | contigs | 1 |
| **MIGS-32** | Relevant SOPs |  |
| **MIGS-33** | Relevant e-resources |  |
